# Beta-Lactam-Resistant *Enterobacterales* Isolated from Landfill Leachates

**DOI:** 10.3390/pathogens11101077

**Published:** 2022-09-22

**Authors:** Alejandra Mondragón-Quiguanas, Miguel Ángel Villaquirán-Muriel, Sandra Patricia Rivera, Doris Rosero-García, Carlos Aranaga, Adriana Correa, Aura Falco

**Affiliations:** 1 Universidad Santiago de Cali, Grupo de Investigación en Microbiología, Industria y Ambiente (GIMIA), Facultad de Ciencias Básicas, Cali 760035, Colombia; 2 Laboratorio de Salud Pública Departamental, Secretaria Departamental de Salud del Valle del Cauca, Gobernación del Valle del Cauca, Cali 760045, Colombia; 3 Universidad Santiago de Cali, Grupo de Investigación en Química y Biotecnología (QUIBIO), Facultad de Ciencias Básicas, Cali 760035, Colombia; 4 Clínica Imbanaco, Cali 760042, Colombia

**Keywords:** landfill leachates, *Enterobacterales*, antibiotic, resistance, beta-lactams, Colombia, *Klebsiella pneumoniae*

## Abstract

Antibiotic resistance is one of the main challenges worldwide due to the high morbidity and mortality caused by infections produced by resistant bacteria. In Colombia, this problem has been studied mainly from the clinical perspective; however, it is scarcely studied in the leachates produced in landfills. The objective of this study was to detect, identify and determine the antibiotic sensitivity profile of *Enterobacterales* isolated from a leachate treatment plant located in Cali, Colombia. Detection was performed using selective culture media, bacterial identification using Matrix-Assisted Laser Desorption/Ionization-Time-Of-Flight (MALDI-TOF, *bioMérieux*) and by sequencing the gene coding for the 16S ribosomal RNA subunit when discrepancies were observed between phenotypic characteristics and MALDI-TOF. Antibiotic sensitivity profiling was determined using the automated VITEK^®^2 system (*bioMérieux*). Twenty-one isolates were obtained, of which *Klebsiella pneumoniae* was the most frequent (23.8%), and 34% of the isolates showed decreased sensitivity to beta-lactam antibiotics such as cefoxitin, ampicillin/sulbactam and piperacillin/tazobactam. These findings suggest that leachates from landfills could be a reservoir of pathogenic bacteria carrying antibiotic resistance determinants, so periodic microbiological characterization of these effluents should be performed, promoting the One Health approach.

## 1. Introduction

A landfill is a space designed for final garbage disposal [1]. According to a World Bank report, in 2050 the global amount of deposits in landfills is estimated to exceed 350 million tons per year, driven by the rapid urbanization and population growth [2]. Navarro landfill was the site for final municipal solid waste disposal in the city of Santiago de Cali, Valle del Cauca Department, for 40 years, and was closed in 2008 [3] due to the pollution of the Cauca River (from which 75% of the Cali population is supplied with drinking water through the treatment plant) and the subsoil, and due to natural fires on landfill grounds [4]. A large volume of leachates has been produced after the closure of the former Navarro landfill (AVN, in Spanish), representing a pollution hazard to soil and surrounding groundwater. As a result, a Leachate Treatment Plant (LTP) was built [3]. Landfill leachates result from the percolation of fluids through a solid and generally carry large amounts of the compounds present in that solid [5]. Landfills receive solid household waste, feces and clinical waste, among others. This stockpile generates a large volume of leachates with traces of antibiotics, turning this liquid into a potential reservoir of antibiotic-resistant bacteria [6].

According to the “One Health” approach, there is a connection between human, animal and environmental health, and it indicates that one of the most relevant areas of work is bacterial resistance to antibiotics [7]. Precisely, leachates can cause problems in human, animal and environmental health because they are known to contain numerous antibiotics, antibiotic-resistant bacteria (ARB) and antibiotic resistance genes (ARGs) [8,9,10,11], as reported by Wu et al., who detected twenty (20) antibiotics and six (6) resistance genes (*sul*1, *sul*2, *tet*Q, *tet*M, *erm*B and *mef*A) in leachates from a landfill in Shanghai, China [12]. These genes were also reported by Liu et al. in leachates from Zhejiang Province, China [13]. In addition, mobile genetic elements (MGE), such as plasmids, transposons and integrons have been identified [14,15,16]. According to Arenas and Melo, bodies of water that contain organic loads could act as important antibiotic-resistant bacteria reservoirs because they favor optimal conditions in terms of humidity, pH, temperature, and nutrient availability [11].

All the above underlines the need to improve our understanding of how antibiotic resistance develops and spreads in humans, animals, and the environment [17]. Although in Colombia these types of reports have focused mainly on the clinical perspective [18] because of a direct relationship between them, in the country there have also been studies of bacterial resistance to antibiotics in rivers and wastewater [19,20], in wastewater treatment plants [21,22,23] and in drinking water [24]. However, there are no studies that have characterized bacteria from landfill leachates and their association with antibiotic resistance. Consequently, the objective of this study was to detect, identify and determine the antibiotic sensitivity profile of *Enterobacterales* isolated from a leachate treatment plant located in Cali, Colombia.

## 2. Results

### 2.1. Isolation of Enterobacterales

Among a total of thirty (30) isolates grown in Chromocult^®^ [25], twenty-one (21) were selected. Their colonies showed coliform phenotype. Two of them had a blue coloring corresponding to *Escherichia coli* (fecal coliform), and the remaining nineteen had a pink coloring, which is associated with total coliforms. The remaining nine colonies had a colorless tone; they were presumably *Pseudomonas* [25], which is why they were not included in this study.

### 2.2. Bacterial Identification

**Figure 1:**
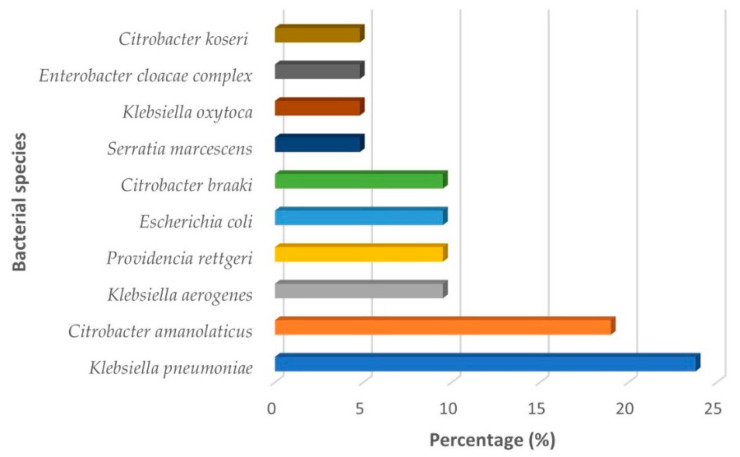
Percentages of *Enterobacterales* identified using MALDI-TOF MS.

Of the twenty-one isolates that were grown on MacConkey agar, 86% (*n* = 18/21) were found to have a lactose-fermenting phenotype. *Klebsiella pneumoniae* had a frequency of 23.8%, followed by *Citrobacter amalonaticus* (19%) (Figure 1). The following Gram-negative bacteria were also identified: *Providencia rettgeri*, *Klebsiella aerogenes*, *E. coli* and *Citrobacter braaki* with frequencies of 9.5% each. *Serratia marcescens*, *Klebsiella oxytoca*, *Enterobacter cloacae* complex and *Citrobacter koseri* had frequencies of 4.8% each (Figure 1).

The identification of one (1) species belonging to the *E. cloacae* complex yielded a 50% confidence level using MALDI-TOF MS. To confirm this match, further identification was performed by 16S ribosomal RNA (16S rRNA) gene sequencing. The isolate showed 99.8% identity with *Enterobacter hormaechei* subsp. *ludwigi*.

### 2.3. Antibiotic Sensitivity Profile Determination

**Figure 2:**
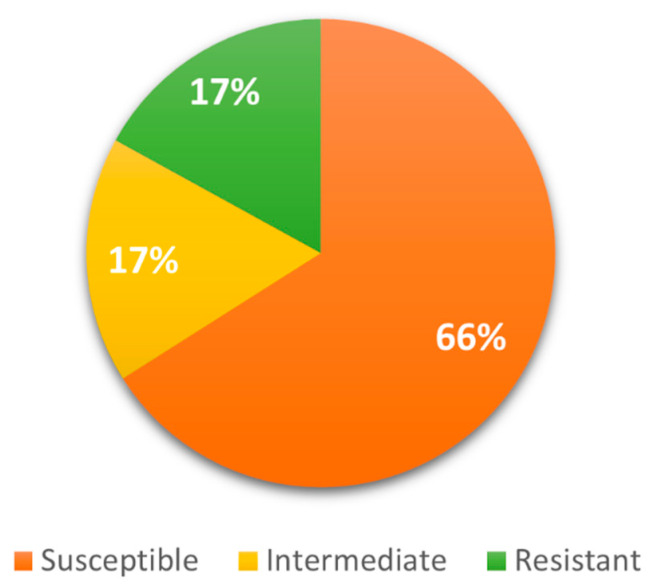
Percentages of sensitivity and resistance to antibiotics in the *Enterobacterales* isolates evaluated.

Sixty-six percent (66%, *n* = 14/21) of the isolates were sensitive to the antibiotics tested (beta-lactams: ceftazidime, ceftriaxone, cefepime, doripenem, ertapenem and meropenem; aminoglycosides: amikacin and gentamicin; ciprofloxacin; and tigecycline), and 34% (*n* = 7/21) presented an intermediate phenotype and/or resistance to beta-lactams (Figure 2).

In general, decreased sensitivity to ampicillin/sulbactam, piperacillin/tazobactam and cefoxitin was observed in seven isolates (Table 1). Regarding the group of beta-lactamase inhibitor antibiotics, decreased sensitivity was observed for ampicillin/sulbactam in isolates Kp2, Kp4 and Pr19; for piperacillin/tazobactam, this was observed in isolates Ka1, Cb2 and Ec1. As for cephamycins, decreased susceptibility to cefoxitin was observed in isolates Ka1, Ka2 and Pr19 (Table 1). Four (*n* = 4/7) of the isolates with intermediate resistance and/or resistance to beta-lactams belonged to the genus *Klebsiella*, and the remaining belonged to the genera *Escherichia*, *Providencia* and *Citrobacter*, with one isolate each (*n* = 1/7).

## 3. Discussion

The transmission routes of Antimicrobial Resistance (AR) are partially known because investigations are predominantly focused on hospital settings, especially the urban setting [26]. However, little is known about the spread of AR in non-clinical settings, especially in Latin America. Due to this, studies have been conducted in which ARGs have been detected worldwide and in various non-clinical settings, including sediments [27,28], activated sludge from wastewater treatment plants [29,30], pig farms [31] and the soil adjacent to them [32], but not in leachates. For this reason, this study describes the resistance to beta-lactam antibiotics in *Enterobacterales* isolated from leachates produced in a treatment plant located in Cali, Colombia, because this group of microorganisms plays an important role in the propagation of antibiotic resistance genes [33].

Historically, unused, or fully consumed antibiotics inevitably end up in landfills through household waste due to the lack of regulation for their disposal [10]. An example of this is that sulfonamides have been detected in landfill leachates both in the state of Florida in the United States [34] and in China [35]. The selective pressure exerted by these drugs on bacteria contributes to their prevalence in landfills and leachates, making them reservoirs [10]. The main concern about leachates produced from landfills is their impact on the surrounding surface and groundwater because, although they have geomembranes, if these are affected by fires or the presence of animals, the environmental impact could be severe [36]. However, there are few studies on ARB in landfill leachates. Threedeach et al. found that *E. coli* isolates from landfill leachates showed resistance to two groups of antibiotics, tetracyclines (37.5–68.8%) and beta-lactams (cephalothin, 57.5–61.3%) [10,37].

In this work we performed a descriptive study, with purposeful sampling via selective and differential culture methods that allowed the selection of *Enterobacterales* from a leachate sample. Regarding bacterial identification, our results indicate that *K. pneumoniae* (23.8%, *n* = 5/21) and *C. amalonaticus* (19%, *n* = 4/21) are the predominant species (Figure 1); and *E. coli* and *E. cloacae* complex have frequencies of 9.5% and 4.8%, respectively (Figure 1). A study by Obire and Guda (2002) [38] demonstrated the presence of species belonging to the genera *Klebsiella*, *Citrobacter*, *Escherichia* and *Enterobacter* in leachates from a landfill in Nigeria, which is consistent with the results found in this study. Although species belonging to these genera have been associated with fecal contamination [39], their presence in highly complex matrices such as leachates is [40] mainly associated with their ability to tolerate and bioremediate heavy-metal-contaminated environments [41,42,43,44,45,46].

Regarding the antibiotic sensitivity profiles, our results indicate that 34% of the isolates showed decreased sensitivity to at least one of the beta-lactam antibiotics tested in this study (Table 1). Beta-lactams are broad-spectrum antibiotics used to treat infections caused by Gram-negative and Gram-positive bacteria [47]. The antibiotic piperacillin/tazobactam is a combination of a beta-lactam and a beta-lactamase inhibitor and is widely used in clinical practice. Isolates of *E. coli* (Eco1), *K. aerogenes* (Ka1) and *C. braaki* (Cb2) species showed resistance to this antibiotic. This resistance may be due to hyperproduction of constitutive chromosomal beta-lactamases such as AmpC [48]. Resistance to this class of antibiotics has also led to the development of inhibitor-resistant (IR) variants, including the TEM and SHV beta-lactamase enzyme families [49]. However, it was a limitation of the study not to be able to detect the genes involved in resistance to these antibiotics.

Bacteria of the genus *Klebsiella* often cause hospital and community acquired urinary tract infections, pneumonia, surgical wound infections, and life-threatening infections, such as endocarditis and septicemia [50]. This bacterial genus has been isolated from landfill leachate samples in Ghana and presented resistance to beta-lactam antibiotics, such as cefuroxime [51]. However, they did not evaluate carbapenems, which are also beta-lactam antibiotics. This study also reports that the genus *Klebsiella* was sensitive to aminoglycosides (gentamicin, amikacin) and quinolones (ciprofloxacin) (Table 1). This is consistent with the results obtained in this study for the genus *Klebsiella* isolates, which also presented sensitivity to gentamicin and resistance and intermediate resistance to amikacin [52].

A *K. aerogenes* isolate (Ka2) presented sensitivity to ertapenem and resistance to imipenem, which is rare (Table 1). This phenotype might be associated with changes in the outer membrane permeability and overexpression of AmpC-type beta-lactamases and efflux pumps, as previously reported [52,53]. Further studies are required to confirm this phenotype. Imipenem is one of the antimicrobial choices for empirical treatment of infections suspected to be caused by extended-spectrum beta-lactamase (ESBL) or AmpC-producing bacteria, which develop resistance to third generation cephalosporins. Imipenem is also used in patients who have been previously treated with broad-spectrum antibiotics due to the possibility of having selected multi-resistant strains [54]. Resistance to these antibiotics is regarded as a global public health problem because carbapenems are used as last-line antibiotics—that is, the ones used against bacteria that show resistance to all other antibiotics.

According to the sensitivity profiles of the rest of the isolates, Kp2, Kp4 and Pr1, it is suggested that the beta-lactam resistance was due to IR production, which should be verified with extra experiments such as specific PCRs for the pre-subject genes involved and/or whole genome sequencing.

The LTP was designed to contain and treat chemical and biological pollutants from the old Navarro landfill. This plant operates by collecting the leachates at the pumping station, from which they are transported and stored in eight lagoons (covered and uncovered) with and without physicochemical treatment. Each lagoon has a geomembrane that isolates it from the surrounding soil and constitutes a complex ecosystem in which various pollutants coexist. This study demonstrated the presence of antibiotic resistant *Enterobacterales* that are potentially pathogenic for both animals and humans in the leachate from the LTP pumping station. These results emphasize the importance of the LTP in reducing microbiological risks that could pollute both the soil and nearby groundwater, which in turn could cause disease outbreaks mainly in the community of Navarro. However, constant monitoring of the microbiomes of the different stations is necessary, since the presence of contaminants such as antibiotics and heavy metals, among others, expose microorganisms to various selective pressures. This has repercussions for the dynamics and genetics of the bacterial community which, through the horizontal transfer of genetic material, could turn this ecosystem into a potential reservoir for the dissemination of antibiotic-resistant bacteria (ARB) and antibiotic resistance genes (ARGs) [14]. Additionally, during the storage of leachates in the lagoons, nearby animals such as birds and rodents, could have contact with leachate bacteria, increasing the risk of zoonotic events, since a large part of the population of Navarro is dedicated to sugar cane agriculture [55]. In line with the present study, opportunistic bacteria were isolated from leachates in the San Nicolás landfill in México [56]. Therefore, it is important to perform frequent surveillance in these environments because if these leachates are not adequately treated, they can impact not only public health (human and animal), but also environmental health.

## 4. Materials and Methods

### 4.1. Collection of Samples

**Figure 3:**
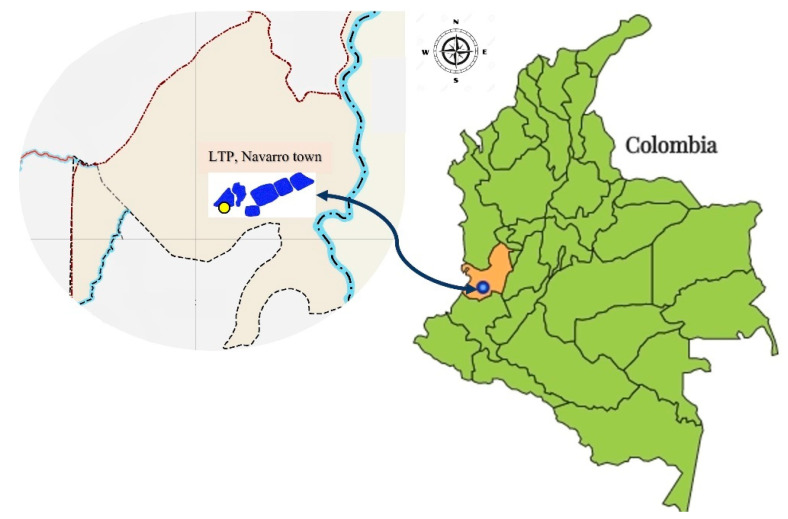
Leachate Treatment Plant (LTP) located in Navarro town, Santiago de Cali municipality (blue dot), Valle del Cauca department (orange), and location of sampling site PS (yellow dot).

This was a descriptive study, with purposeful sampling in selective and differential culture media that allowed the selection of *Enterobacterales* from a sample of leachates collected at a LPT in the town of Navarro (3°23′13.8″ N 76°29′7.5″ W), 2.2 miles from the city of Santiago de Cali. The treatment plant consists of a Pumping Station (PS), from which the sample was taken, which distributes the leachate from the landfill to eight lagoons, five of which are treated by physicochemical methods, and remaining three are untreated (Figure 3). The sample was collected in March 2019.

### 4.2. Isolation of Enterobacterales

From the landfill leachate sample, two aliquots of 100 mL were collected using a sterile 500 mL glass vial and then filtered through a 0.45 µm cellulose ester membrane (Merck Millipore, Darmstadt, Germany) using a vacuum filtration equipment (Merck Millipore, Germany). Each filter was transferred to Chromocult Agar^®^ (Merck Millipore, Germany) [25], and MacConkey Agar (Oxoid, Basingstoke, UK). The plates were incubated for 18–20 h at 37 °C. Colonies of presumptive *Enterobacterales* were picked up from the plates and restraeked on Soy Trypticase Agar (STA) (Oxoid, Basingstoke, UK) plates to obtain pure cultures.

### 4.3. Bacteria Identification

All isolates were grown for 18–20 h in STA for identification by MALDI-TOF MS (VITEK^®^MS *bioMérieux*, Durham, NC, USA), following the manufacturer’s recommendations.

When the confidence level obtained with MALDI-TOF MS was less than 80%, the isolate was identified using 16S ribosomal RNA (16S rRNA) gene sequencing. For this, the bacterial strain was grown on Trypticase Soy Broth (TSB) (Oxoid, UK) and incubated for 18 h at 37 °C. Then, 200 µL of the culture were added to 800 µL of distilled water and boiled for 10 min. The bacterial suspension was then centrifuged at 12,000 rpm for 5 min, and the supernatant was used for PCR. The 16S rRNA gene was amplified by PCR using primers U1 and U2, previously described in [57], and PCR 100 2X Master Mix (CorpoGen, OPTBM-00006, Bogotá, Colombia). After PCR amplification, fragments were purified with Qiaquick PCR Spin columns (Qiagen, Hilden, Germany) and sequenced in both forward and reverse directions (Macrogen, Seoul, Korea) with the same primers used for PCR amplification. The sequences were compared with the National Center for Biotechnology Information (NCBI) (https://blast.ncbi.nlm.nih.gov/Blast.cgi (accessed on 21 April 2022)) and The Ribosomal Database Project (https://rdp.cme.msu.edu/ ( accessed on 21 April 2022)).

### 4.4. Determination of Antibiotic Sensitivity Profiles

The determination of the antibiotic sensitivity profiles of the bacterial isolates was performed using the automated system VITEK^®^2 (*bioMérioux*, Durham, NC, USA), using the VITEK^®^ N272-AST card (reference 414164, *bioMérieux*, Durham, NC, USA), following the manufacturer’s recommendations.

## 5. Conclusions

In this study, potentially pathogenic species for humans and animals belonging to the order *Enterobacterales* were isolated, with *K. pneumoniae* being the most prevalent bacterium (23.8%), followed by *Citrobacter amalonaticus* (19%). Although there was a low frequency of beta-lactam-resistant bacteria and no multidrug-resistant isolates were detected, they could act as a reservoir of resistance genes in leachates and are therefore considered a public health and environmental concern. Therefore, research on ARs in landfills and landfill leachates is essential to better understand the mechanism of transport and distribution of ARs in the environment. To our knowledge, this is the first study of antibiotic resistance in leachates in Colombia, which is why the findings obtained could be useful for policy makers on this critical issue in the country.

## Figures and Tables

**Table 1:** Antimicrobial susceptibility profiles of strains belonging to *Enterobacterales*.

		MIC (µg/mL) Per Antibiotic/Interpretative Categories													
Strain ID	BLEE	SAM ^1^	TZP ^1^	FOX ^2^	CAZ ^3^	CRO ^3^	FEP ^3^	DOR ^4^	ETP ^4^	IPM ^4^	MEM ^4^	AMK ^5^	GEN ^5^	CIP ^6^	TIG ^7^
Kp1	Negative	8 (S)	≤4 (S)	≤4 (S)	≤1 (S)	≤1 (S)	≤1 (S)	≤0.12 (S)	≤0.5 (S)	≤0.25 (S)	≤0.25 (S)	≤2 (S)	≤1 (S)	≤0.25 (S)	≤0.5 (S)
Kp2	Negative	16 (I)	≤4 (S)	8 (S)	≤1 (S)	≤1 (S)	≤1 (S)	≤0.12 (S)	≤0.5 (S)	≤0.25 (S)	≤0.25 (S)	≤2 (S)	≤1 (S)	≤0.25 (S)	1 (S)
Kp3	Negative	8 (S)	≤4 (S)	≤4 (S)	≤1 (S)	≤1 (S)	≤1 (S)	≤0.12 (S)	≤0.5 (S)	≤0.25 (S)	≤0.25 (S)	≤2 (S)	≤1 (S)	≤0.25 (S)	≤1 (S)
Kp4	Negative	≥32 (R)	8 (S)	≤4 (S)	≤1 (S)	≤1 (S)	≤1 (S)	≤0.12 (S)	≤0.5 (S)	≤0.25 (S)	≤0.25 (S)	≤2 (S)	≤1 (S)	≤0.25 (S)	≤0.5 (S)
Kp5	Negative	8 (S)	≤4 (S)	≤4 (S)	≤1 (S)	≤1 (S)	≤1 (S)	≤0.12 (S)	≤0.5 (S)	≤0.25 (S)	≤0.25 (S)	≤2 (S)	≤1 (S)	≤0.25 (S)	1 (S)
Ka1	Negative	4 (S)	64 (I)	≥64 (R)	≤1 (S)	≤1 (S)	≤1 (S)	≤0.12 (S)	≤0.5 (S)	1 (S)	≤0.25 (S)	≤2 (S)	≤1 (S)	≤0.25 (S)	1 (S)
Ka2	Negative	4 (S)	≤4 (S)	≥64 (R)	≤1 (S)	≤1 (S)	≤1 (S)	≤0.12 (S)	≤0.5 (S)	2 (I)	≤0.25 (S)	≤2 (S)	≤1 (S)	≤0.25 (S)	≤0.5 (S)
Ko1	Negative	8 (S)	≤4 (S)	≤4 (S)	≤1 (S)	≤1 (S)	≤1 (S)	≤0.12 (S)	≤0.5 (S)	0.5 (S)	≤0.25 (S)	≤2 (S)	≤1 (S)	≤0.25 (S)	≤0.5 (S)
Sm1	N/A	NR	≤4 (S)	NR	≤1 (S)	≤1 (S)	≤1 (S)	0.25 (S)	≤0.5 (S)	1 (S)	≤0.25 (S)	≤2 (S)	≤1 (S)	≤0.25 (S)	1 (S)
Ca1	N/A	N/A	≤4 (S)	NR	≤1 (S)	≤1 (S)	≤1 (S)	≤0.12 (S)	≤0.5 (S)	≤0.25 (S)	≤0.25 (S)	≤2 (S)	≤1 (S)	≤0.25 (S)	≤0.5 (S)
Ca2	N/A	N/A	≤4 (S)	NR	≤1 (S)	≤1 (S)	≤1 (S)	≤0.12 (S)	≤0.5 (S)	≤0.25 (S)	≤0.25 (S)	≤2 (S)	≤1 (S)	≤0.25 (S)	≤0.5 (S)
Ca3	N/A	N/A	≤4 (S)	NR	≤1 (S)	≤1 (S)	≤1 (S)	≤0.12 (S)	≤0.5 (S)	≤0.25 (S)	≤0.25 (S)	≤2 (S)	≤1 (S)	≤0.25 (S)	≤0.5 (S)
Ca4	N/A	N/A	≤4 (S)	NR	≤1 (S)	≤1 (S)	≤1 (S)	0.25 (S)	≤0.5 (S)	1 (S)	≤0.25 (S)	≤2 (S)	≤1 (S)	≤0.25 (S)	1 (S)
Cb1	N/A	N/A	≤4 (S)	NR	≤1 (S)	≤1 (S)	≤1 (S)	≤0.12 (S)	≤0.5 (S)	1 (S)	≤0.25 (S)	≤2 (S)	≤1 (S)	≤0.25 (S)	≤0.5 (S)
Cb2	N/A	N/A	≥128 (R)	NR	≤1 (S)	≤1 (S)	≤1 (S)	≤0.12 (S)	≤0.5 (S)	≤0.25 (S)	≤0.25 (S)	≤2 (S)	≤1 (S)	≤0.25 (S)	≤0.5 (S)
Ck1	N/A	N/A	≤4 (S)	NR	≤1 (S)	≤1 (S)	≤1 (S)	≤0.12 (S)	≤0.5 (S)	≤0.25 (S)	≤0.25 (S)	≤2 (S)	≤1 (S)	≤0.25 (S)	≤0.5 (S)
Ec1	Negative	4 (S)	64 (I)	≤4 (S)	≤1 (S)	≤1 (S)	≤1 (S)	≤0.12 (S)	≤0.5 (S)	≤0.25 (S)	≤0.25 (S)	≤2 (S)	≤1 (S)	≤0.25 (S)	≤0.5 (S)
Ec2	Negative	8 (S)	≤4 (S)	≤4 (S)	≤1 (S)	≤1 (S)	≤1 (S)	≤0.12 (S)	≤0.5 (S)	≤0.25 (S)	≤0.25 (S)	≤2 (S)	≤1 (S)	≤0.25 (S)	1 (S)
Pr19	N/A	≥32 (R)	≤4 (S)	16 (I)	≤1 (S)	≤1 (S)	≤1 (S)	N/A	≤0.5 (S)	1 (S)	≤0.25 (S)	≤2 (S)	≤1 (S)	≤0.25 (S)	NR
Pr20	N/A	8 (S)	≤4 (S)	≤4 (S)	≤1 (S)	≤1 (S)	≤1 (S)	N/A	≤0.5 (S)	2 (S)	≤0.25 (S)	2 (S)	≤1 (S)	≤0.25 (S)	NR
Ecc1	N/A	NR	≤4 (S)	NR	≤1 (S)	≤1 (S)	≤1 (S)	≤0.12 (S)	≤0.5 (S)	1 (S)	≤0.25 (S)	≤2 (S)	≤1 (S)	≤0.25 (S)	1 (S)
Ec ATCC 25922	≤2 (S)	≤4 (S)	≤4 (S)	≤1 (S)	≤1 (S)	≤1 (S)	≤0.12 (S)	≤0.5 (S)	≤0.25 (S)	≤0.25 (S)	≤2 (S)	≤2 (S)	≤1 (S)	≤0.5 (S)	≤0.5 (S)

Abbreviations: MIC, minimal inhibitory concentration; BLEE, extended spectrum ß-lactamases; SAM, ampicillin/sulbactam; TZP, piperacillin/tazobactam; FOX, cefoxitin; CAZ, ceftazidime; CRO, ceftriaxone; FEP, cefepime; DOR, doripenem; ETP, ertapenem; IPM, imipenem; MEM, meropenem; AMK, amikacin; GEN, gentamicin; CIP, ciprofloxacin; TIG, tigecycline; N/A; not applicable; NR: natural resistance; Kp, *K. pneumoniae*; Ka, *Klebsiella aerogenes*; Ko, *Klebsiella oxytoca*; Sm, *Serratia marcescens*; Ec, *Escherichia coli*; Ca, *Citrobacter amanolaticus*; Cb, *Citrobacter braaki*; Ck, *Citrobacter koseri*; Ecc, *Enterobacter cloacae complex*; Pr, *Providencia rettgeri*; R (dark grey), resistant; I (light gray), intermediate; S, susceptible; NEG, negative; ^1^—penicillin + β-lactamase inhibitors; ^2^—cephamycins; ^3^—extended-spectrum cephalosporins; ^4^—carbapenems; ^5^—aminoglycosides; ^6^—fluoroquinolones; ^7^—tetracycline. Shadows highlight bacterial resistance to tested antibiotics.

## Data Availability

The data used to support the findings of this study are included within the article and are available from the corresponding author upon request.
